# High-Frequency Intraoral Ultrasound for Preoperative Assessment of Depth of Invasion for Early Tongue Squamous Cell Carcinoma: Radiological–Pathological Correlations

**DOI:** 10.3390/ijerph192214900

**Published:** 2022-11-12

**Authors:** Simone Caprioli, Alessandro Casaleggio, Alberto Stefano Tagliafico, Cristina Conforti, Fabio Borda, Martina Fiannacca, Marta Filauro, Andrea Iandelli, Filippo Marchi, Giampiero Parrinello, Giorgio Peretti, Giuseppe Cittadini

**Affiliations:** 1 Department of Internal Medicine (DIMI), University of Genova, Viale Benedetto XV 6, 16132 Genoa, Italy; 2 Department of Radiology, IRCCS Ospedale Policlinico San Martino, Largo Rosanna Benzi 10, 16132 Genoa, Italy; 3 Department of Health Sciences (DISSAL), University of Genova, Via Pastore 1, 16132 Genoa, Italy; 4 Department of Otorhinolaryngology, Head and Neck Surgery, IRCCS Ospedale Policlinico San Martino, Largo Rosanna Benzi 10, 16121 Genoa, Italy; 5 Department of Surgical Sciences and Integrated Diagnostics (DISC), University of Genoa, 16132 Genoa, Italy; 6 Department of Experimental Medicine (DIMES), University of Genoa, 16132 Genoa, Italy

**Keywords:** tongue squamous cell carcinoma, depth of invasion, intraoral ultrasound

## Abstract

The eighth edition of the TNM classification officially introduced “depth of invasion” (DOI) as a criterion for determining the T stage in tongue squamous cell carcinoma. The DOI is a well-known independent risk factor for nodal metastases. In fact, several experts strongly suggest elective neck dissection for tongue cancer with a DOI > 4 mm due to the high risk of early and occult nodal metastases. Imaging plays a pivotal role in preoperative assessments of the DOI and, hence, in planning the surgical approach. Intraoral ultrasound (IOUS) has been proposed for early-stage SCC of the oral tongue as an alternative to magnetic resonance imaging (MRI) for local staging. The aim of this work is to investigate the accuracy of IOUS in the assessment of the DOI in early oral SCC (CIS, pT1, and pT2). A total of 41 patients with tongue SCCs (CIS-T2) underwent a preoperative high-frequency IOUS. An IOUS was performed using a small-size, high-frequency hockey-stick linear probe. The ultrasonographic DOI (usDOI) was retrospectively compared to the pathological DOI (pDOI) as the standard reference. In patients who underwent a preoperative MRI, their usDOI, magnetic resonance DOI (mriDOI), and pDOI were compared. Specificity and sensitivity for the IOUS to predict a pDOI > 4 mm and to differentiate invasive and noninvasive tumors were also evaluated. A high correlation was found between the pDOI and usDOI, pDOI and mriDOI, and usDOI and mriDOI (Spearman’s ρ = 0.84, *p* < 0.0001, Spearman’s ρ = 0.79, *p* < 0.0001, and Spearman’s ρ = 0.91, *p* < 0.0001, respectively). A Bland–Altman plot showed a high agreement between the usDOI and pDOI, even though a mean systematic error was found between the usDOI and pDOI (0.7 mm), mriDOI and pDOI (1.6 mm), and usDOI and mriDOI (−0.7 mm). The IOUS was accurate at determining the T stage (*p* < 0.0001). The sensitivity and specificity for the IOUS to predict a pDOI ≥4 mm were 92.31% and 82.14%, respectively, with an AUC of 0.87 (*p* < 0.0001). The specificity, sensitivity, negative predictive value (NPV), and positive predictive value (PPV) for the IOUS to predict an invasive cancer were 100%, 94.7%, 60%, and 100%, respectively. The AUC was 0.8 (95% CI 0.646–0.908, *p* < 0.0001). The IOUS was accurate in a preoperative assessment of a pDOI and T stage, and can be proposed as an alternative to MRI in the preoperative staging of tongue SCC.

## 1. Introduction

**Figure 1:**
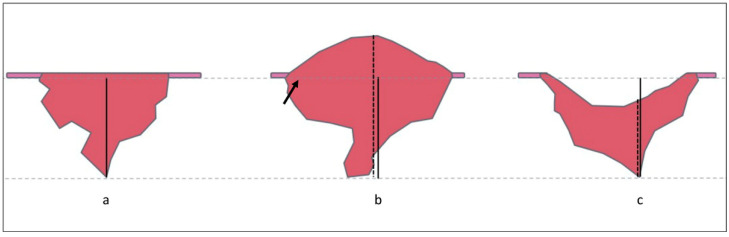
Cartoon demonstrating how to measure DOI in flat (**a**), exophytic (**b**), or ulcerated lesions (**c**). The reference line is the basal membrane. DOI is measured perpendicularly to the basal membrane, including ulcerated parts of the lesion, but excluding the exophytic portions. Tumor thickness is greater than DOI in exophytic lesions and smaller in ulcerated neoplasms. Solid line = DOI; dashed line = tumor thickness; arrow = basal membrane.

Oral squamous cell carcinoma (SCC) is the most frequent head and neck neoplasm, and the oral tongue is the most common site of presentation [1]. Despite innovations in treatment, the prognosis of tongue SCC is still difficult to predict: even though overall survival and disease-specific survival are satisfactory at early stages, the risk of lymphatic dissemination is high and represents the most critical prognostic factor [2]. Moreover, the prevalence of occult nodal metastasis in a clinically negative neck ranges from 8.2% to 46.3%, and mortality is increased five-fold if occult nodal metastases occur [2,3,4,5]. In 2017, the eighth edition of the tumor–node–metastasis (TNM) staging system of the American Joint Cancer Committee (AJCC)/Union for International Cancer Control (UICC) officially introduced the depth of invasion (DOI) as a staging criterion for the T stage along with the surface dimension in oral SCC [6]. The DOI is defined as the depth of the tumor invasion measured from the level of the basement membrane of the closest normal mucosa following an ideal “plumb line” [7] (Figure 1).

The DOI is a well-known prognostic factor. In addition, several authors have demonstrated a direct correlation between the DOI and the incidence of nodal metastasis [2,3,8,9]. For these reasons, elective neck dissection has been proposed for early-stage oral tongue SCC. Furthermore, the DOI can be used to dictate prophylactic neck dissection in clinically N0 patients. Notwithstanding, there is no global consensus for the threshold, with a range varying between 3 mm and 10 mm [3,10,11]. However, a DOI cut-off of 4 mm was recently proposed by several experts [12,13,14].

Imaging plays a pivotal role in the local staging of tongue cancer by estimating the DOI and guiding the surgeon to plan the surgical intervention properly and define the need for an elective neck dissection. In fact, cT1 and selected cT2 can be safely removed transorally. In contrast, more advanced tumors require a pull-through approach to obtain better control of deep margins [15]. Computed tomography, magnetic resonance imaging (MRI), and an intraoral ultrasound (IOUS) can be used to assess the local and regional extent of oral cancer (Table 1). MRI is now considered the first choice for the preoperative staging of oral cancer [16], but the interest in IOUS has been progressively growing over time. Several studies have reported the utility of an IOUS in the preoperative staging of oral tongue SCC [17]. In an early study, Shintani and coworkers compared IOUS with CT and MRI using histology as the gold standard. They reported that ultrasonography was superior to CT and MRI in an assessment of the primary lesion of oral carcinoma, mostly because CT and MRI could not detect the primary tumor if the thickness was less than 5 mm [18]. IOUS is also used for intraoperative tumor thickness assessments and to improve locoregional control during surgery [19,20]. However, most studies in the literature evaluated IOUS in the assessment of tumor thickness [21,22,23,24,25,26,27,28,29,30].

Although conceptually different, tumor thickness (TT) and the DOI demonstrated a good prognostic performance due to the high correlation with the risk of lymph node metastases. However, the use of TT instead of the DOI can cause the risk of upstaging in a small percentage of patients; moreover, the DOI is now considered to be the most reliable parameter to predict the risk of lymph node metastases and prognosis [31,32]. Only four studies have investigated the role of an IOUS in the assessment of the DOI to date. Iida and colleagues demonstrated a good correlation between an ultrasonographic DOI (usDOI) and pathological DOI (pDOI), even in early tongue SCCs (<5 mm) [33]. Filauro and coworkers compared the usDOI and MRI-measured DOI (mriDOI) and demonstrated a better correlation between the mriDOI and pDOI than between the usDOI and pDOI [34]. Rocchetti and colleagues found a strong correlation between the usDOI and pDOI, but a moderate correlation between the usDOI and US-measured diameter; moreover, they found a high sensitivity, specificity, and PPV in the assessment of the infiltration of the tumor beyond the lamina propria into the submucosa (93.1%, 100%, and 100%, respectively) [35]. Takamura and coworkers found a high radiological–pathological agreement and showed that an IOUS was more accurate than CT and MRI at detecting T1 and T2 in squamous cell carcinomas [36]. To date, few studies have analyzed the role of IOUSs in the preoperative staging of early tongue SCC [22,23,24,26,28,35,37], but only the study by Takamura et al. evaluated the radiological–pathological agreement between the pDOI and usDOI [36]. Herein, we assessed if the IOUS could be an alternative staging tool, especially for early SCC. In particular, we investigated the ability of the IOUS to predict the pDOI and T stage in oral tongue SCC, as well as to predict a pDOI > 4 mm, which is the threshold value to perform elective neck dissection in tongue SCC.

## 2. Materials and Methods

### 2.1. Patients

A total of 72 patients underwent an IOUS for oral tongue mucosal lesions from 2017 to 2021 at our institution. The inclusion criteria for the study were: (1) pathologically demonstrated tongue or tongue pelvis SCC; (2) a usDOI assessment; (3) complete surgical excision and histopathological measurement of the DOI. The exclusion criteria were: (1) benign lesion, dysplasia, nonsquamous cell carcinomas; (2) diagnosis of distant metastases and/or synchronous head and neck SCC; (3) treatment with neoadjuvant therapy; (4) T3 to T4 SCC. Among those who met the inclusion criteria for the study, 29 also underwent preoperative MRI.

All patients had been submitted to surgery after a multidisciplinary team (MDT) discussion and preoperative counseling between head and neck surgeons, radiologists, radiation, and medical oncologists. All patients were preoperatively evaluated by a dedicated head and neck surgeon by rigid endoscopy under white light (WL) and narrow-band imaging for the assessment of the superficial boundaries of the lesion.

### 2.2. Measurement of Radiological and Pathological DOI

**Figure 2:**
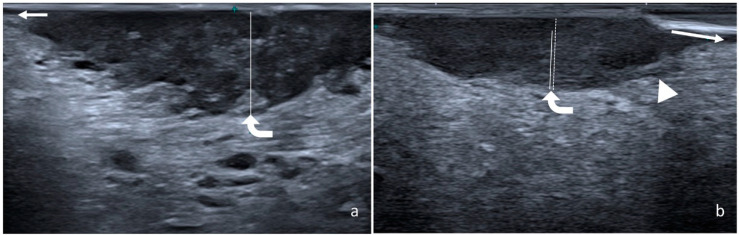
Two cases of squamous cell carcinoma of the lateral surface of the tongue. Flat (**a**) and exophytic (**b)** lesions are shown. In each image, DOI (solid line) was measured perpendicularly from the mucosal surface to the deepest point of infiltration (curved arrow) using the closest normal mucosa as reference line (arrow). The exophytic portion of lesions (**b**) was not included and consequently DOI (solid line) and tumor thickness (dashed line) were significantly different. Arrowhead: superior longitudinal muscle.

An IOUS was performed using a 22-8 MHz 8 mm footprint hockey-stick probe; for patients who were scanned before 2018, a hockey-stick 15-7 MHz probe was used. The probe was shielded with a latex cover on which a small amount of ultrasound gel was introduced. The examination was performed with the patient extending the tongue, which was gently held with gauze on the contralateral side by the operator. The ultrasound examination was performed using light pressure to avoid compression distortion. The entire lesion was examined to determine the deepest point of infiltration. The usDOI was measured perpendicularly to the mucosal surface using the closest normal mucosa as the reference line; the exophytic parts of the lesion were excluded from the measurement, while the ulcerated part was included (Figure 2).

MRI was performed with a 1.5 T scanner and a 3.0 T scanner, with the manufacturer’s phased-array head and neck coils. The exams were conducted on axial and coronal planes using turbo spin echo (TSE) T1 and T2 weighted sequences, and diffusion-weighted imaging sequences (b-values: 50, 800) with an apparent diffusion coefficient map and fat-saturated gadolinium-enhanced gradient echo T1 weighted sequences. If needed, T1 and T2 weighted sequences for motion artifact reduction were used (using, for instance, radial sampling of the k-space sequences).

The pDOI was assessed using a micrometer in formalin-fixed paraffin-embedded specimens. The DOI was measured following a reference line perpendicular to the plane of the basement membrane of the closest normal mucosa.

### 2.3. Statistical Analyses

Statistical analyses were performed using MedCalc software. A Shapiro–Wilk test was used to study the distribution of the variables. A correlation analysis was performed with Spearman’s rank correlation. The agreement between the pDOI, usDOI, and mriDOI was assessed with the Bland–Altman plot. An χ² test was used to evaluate the ability of the IOUS to correctly assign the tumor in the corresponding pathological T stage (pT stage), testing the null hypothesis that there is no correlation between the ultrasonographically assessed T stage (usT stage) and pT stage. The specificity, sensibility, and area under the ROC curve were calculated to determine the ability of the IOUS to predict a pDOI ≥ 4 mm. Specificity, sensibility, and area under the ROC curve were also assessed for the IOUS to predict the deep invasion beyond the epithelial layer (pT1 and pT2 vs. CIS).

## 3. Results

Altogether, 41 patients met the criteria for the study and were included. Clinicodemographic data are shown in Table 2. The primary site was the lateral surface in 33 cases, ventral surface in 3 cases, dorsal surface in 2 cases, and tongue pelvis in 3 cases. On the US, the tongue SCC appears as a slightly hypoechoic lesion that replaces the normal epithelial layer, which is very hypoechoic. If invasive, it infiltrates the deeper hyperechoic layer, which may represent the subepithelial connective tissue and possibly the intrinsic muscle of the tongue layer.

The mean usDOI, mriDOI, and pDOI were 3.79 mm (95% CI 2.93–4.65), 5.30 mm (95% CI 4.23–6.37), and 3.07 mm (95% CI 2.24–3.91 mm), respectively. The mean difference between the radiological and pathological DOI was 1.06 mm and 1.64 mm for the usDOI and mriDOI, respectively. However, the difference was not statistically significant (*p* = 0.26).

**Figure 3:**
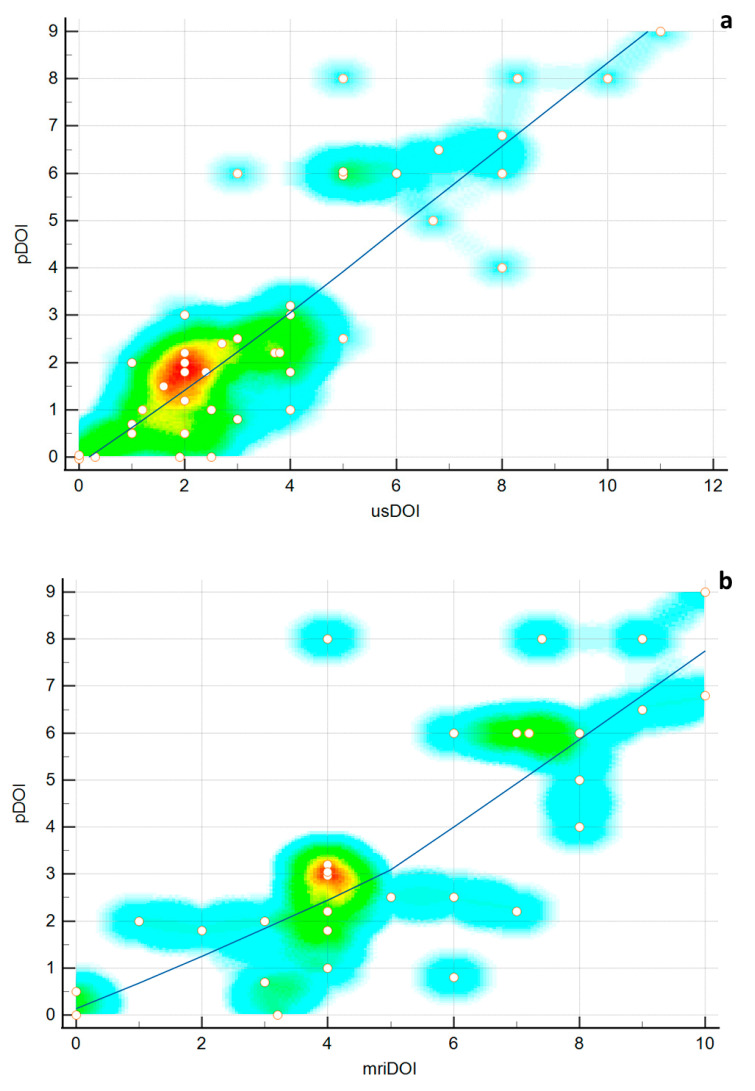
Scatter plots showing the correlation between usDOI and pDOI (**a**) and between mriDOI and pDOI (**b**).

The spearman rank correlation between the pDOI and usDOI, pDOI and mriDOI, and usDOI and mriDOI was 0.84 (95% CI 0.72–0.91, *p* < 0.0001), 0.79 (95% CI 0.60–0.90, *p* < 0.0001), and 0.91 (95% CI 0.80–0.95, *p* < 0.0001), respectively (Figure 3). No statistically significant difference was found.

**Figure 4:**
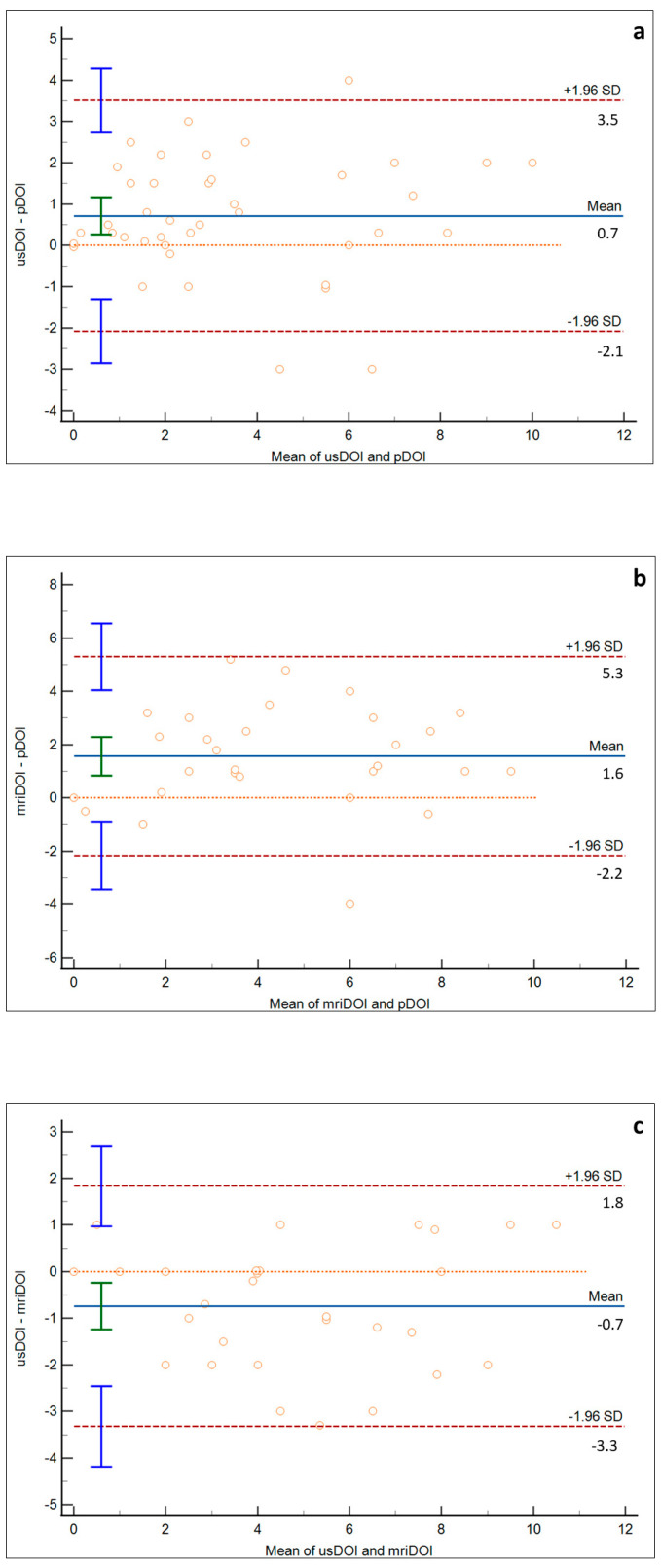
Bland-Altman plots comparing usDOI and pDOI (**a**), mriDOI and pDOI (**b**), and usDOI and mriDOI (**c**). Mean bias between usDOI and pDOI is 0.7 mm, between mriDOI and pDOI is 1.6 mm, and between usDOI and mriDOI is −0.7 mm. Interval agreement is smaller in the usDOI-pDOI plot than in the mriDOI-pDOI plot (red lines: interval agreement; blue line mean difference: orange line: zero line).

Bland–Altman plots between the usDOI and pDOI, mriDOI and pDOI, and usDOI and mriDOI are shown in Figure 4a–c, respectively. The mean bias between the usDOI and pDOI was 0.7 mm (95% CI 0.26–1.16), whereas the bias between the mriDOI and pDOI was 1.6 mm (95% CI 1.56–1.90). The mean bias between the usDOI and mriDOI was −0.7 mm (95% CI −1.24–0.24).

**Figure 5:**
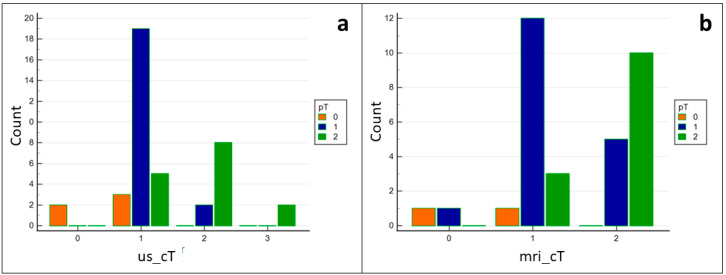
Frequency charts for clinically and pathologically derived T stage. (**a**) = us-derived and pathologically derived T stage. (**b**) = mri-derived and pathologically derived T stage.

**Figure 6:**
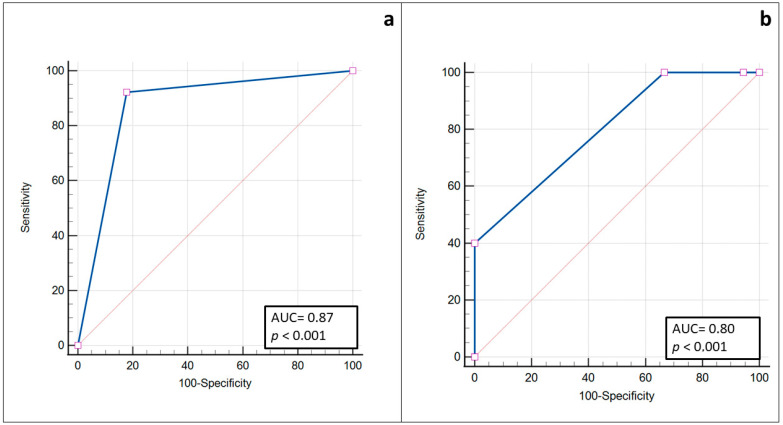
Receiver operating characteristic (ROC) curve for the IOUS assessment of a pDOI > 4 mm (**a**) and for differentiating invasive and noninvasive (carcinoma in situ) tumors (**b**) (blue line: ROC curve; red line: random classifier line).

The null hypothesis that there is no correlation between the usT stage and pT stage was rejected, and the alternative hypothesis that there is a relation between the two classifications was accepted (*p* < 0.0001) (Figure 5). The sensitivity and specificity for the IOUS to predict a pDOI ≥4 mm were 92.31% and 82.14%, respectively. The area under the ROC curve (AUC) was 0.87 (95% CI 0.73–0.95, *p* < 0.0001) (Figure 6).

Using the IOUS, we found that 39 tumors went beyond the epithelial layer and infiltrated the subepithelial connective tissue, while 3 did not. Upon a histological examination, we found that 36 tumors were invasive while 5 were in situ. The specificity, sensitivity, negative predictive value (NPV), and positive predictive value (PPV) for the IOUS to predict an invasive cancer were 100%, 94.7%, 60%, and 100%, respectively. The AUC was 0.8 (95% CI 0.646–0.908, *p* < 0.0001) (Figure 6).

## 4. Discussion

The tongue is the most common subsite for oral SCC [1]. The eighth edition of the TNM classification officially introduced the DOI as a criterion for determining the T stage in oral cancer. The DOI is a well-known prognostic factor. Indeed, several authors have demonstrated a direct correlation between the DOI and the incidence of nodal metastasis [2,3,8,9]. A precise preoperative measurement of the DOI is crucial for planning the surgical approach: cT1 and selected cT2 tumors with a DOI < 10 mm that do not infiltrate extrinsic tongue muscles can be removed transorally [15]. Moreover, if a reliable radiological tool for estimating DOI was available, it would be possible to plan a primary tumor removal and elective neck dissection simultaneously.

Currently, there is no standard radiological technique to estimate the DOI. MRI is considered the first-choice imaging modality for preoperative staging for tongue SCC. However, ultrasonography may be a suitable alternative, thanks to its high spatial resolution, especially in early tumors.

In our study, an excellent radiological–pathological agreement in estimating the DOI was found. Compared with histopathology, both ultrasonography and MRI showed a good correlation, with slightly better performance for ultrasonography than MRI (0.84 and 0.79, respectively), even though the difference was not statistically significant.

The radiological–pathological agreement was studied using the Bland–Altman plot (setting histology as the reference standard), which enabled us to reveal the systematic mean bias. Both ultrasonography and MRI had a good performance since only three cases and one case were outside the limits of agreement, respectively, for the first and second methodology. Not surprisingly, a systematic error was found for both imaging modalities. However, ultrasonography showed a smaller bias and 95% limits of agreement than MRI: the IOUS systematically overestimated the DOI by 0.7 mm, while the mean bias for MRI was 1.6 mm. Therefore, ultrasonography was more precise than MRI with regard to CIS, T1, and T2 tumors. Only the study by Takamura et al. studied radiological–pathological agreement and mean bias between usDOI and pDOI, showing a higher reliability of IOUS than MRI in the preoperative prediction of DOI for T1 and T2 SCCs and a 0.2 mm mean bias between the usDOI and pDOI [36]. The radiological overestimation of the DOI may be related to two phenomena. Firstly, tissue shrinkage has been demonstrated on formalin-fixed surgical specimens of oral SCC [38,39]; moreover, tumors are usually surrounded by peritumoral inflammation and edema that may influence radiological measurements of the DOI, as several authors have demonstrated that MRI significantly overestimates the DOI [40]. Based on our results, we can hypothesize that an IOUS may be less influenced by peritumoral edema. Moreover, for early tongue SSC, an mriDOi mean bias >1 mm has to be considered clinically meaningful.

However, despite the mean bias, our study showed the good performance of the IOUS in estimating the T stage correctly: 70.7% of patients were assigned to the correct pathological T stage, 17.1% of patients were assigned to a higher T stage, and 12.2% of patients were assigned to a lower T stage.

The DOI was found to be a good predictor for occult nodal metastases. Controversies remain about the proper pDOI threshold for a clinically relevant risk of occult nodal metastases; however, data from the literature suggest that an elective neck dissection should be performed with a pDOI ≥ 4 mm [12,13,14]. An accurate instrument to preoperatively measure the DOI may allow proposing concomitant tumor resection and elective neck dissection, reducing the rate of two-step procedures. In our series of early tongue SCCs, the IOUS was very accurate in determining whether a tumor had a DOI ≥ 4 mm or not, which was the threshold chosen for elective neck dissection at our institution, regardless of the T stage, with a sensitivity of 92.31%, specificity of 82.14%, and AUC of 0.87 (95% CI 0.73–0.95, *p* < 0.0001). However, further studies with a larger sample are required to understand if the usDOI alone can be considered as a predictor for neck nodal metastasis and local recurrence in the same way as the pDOI.

One of the main strengths of our work is the use of a very high frequency (22-8 MHz) linear probe, in contrast to most studies in the literature [19,20,21,22,23,24,25,26,27,28,30,33,35,36,37]. Moreover, a small probe allowed a relatively easy intraoral approach, even though it is more difficult to scan posteriorly located tumors. Furthermore, in this study, the use of a very high frequency probe enabled us to recognize the superficial layers of the mucosa of the tongue and, in such a way, to measure the usDOI from the subepithelial connective tissue to the deepest point of infiltration of the tumor. However, caution has to be used for the usDOI assessment of more advanced SCCs: ultrasonography is less reliable if the pDOI is >5 mm [33,41]. For small high-frequency probes, the risk may be even higher, both for the weak penetration of the ultrasound and the small field of view.

The present work enlightens the role ultrasonography in early tongue SCCs. The clinical importance to precisely estimate the DOI in early stages is due to the fact that oral tongue SCCs have an early lymphatic spread and a high risk of occult metastases [2,3,4], especially if they have a DOI > 4 mm. To our knowledge, this is the second study to investigate radiological–pathological agreement in early tongue SCCs as a target and to include CISs, confirming the results in the literature [35,36]. Therefore, we believe that a high-frequency IOUS may be a radiological tool to noninvasively estimate the risk of the invasiveness of a clinically revealed lesion. Thanks to its highest spatial resolution among radiological devices, ultrasonography appears to be indicated explicitly to study small, superficial lesions. Recent work by Rocchetti et al. showed that the sensitivity, specificity, PPV, and NPV of an IOUS in the assessment of the extension of the tumor beyond the lamina propria into the submucosa were 93.1%, 100%, 100%, and 60%, respectively [35]. We report a sensitivity, specificity, PPV, and NPV for the IOUS to predict an invasive cancer of 94.7%, 100%, 100%, and 60%, respectively, in accordance with the literature. However, similar to the study by Rocchetti et al., we only evaluated five carcinomas in situ. Further studies are required to establish the role of an IOUS in differentiating invasive and noninvasive tumors.

The main limitations of this study are the small sample size and retrospective methodology. Moreover, a high-frequency IOUS is a new and operator-dependent technique, and radiologists need time to learn how to measure the DOI correctly.

The sample was too small and the follow-up was insufficient to identify the correlation between the DOI and nodal metastases. However, since we found a high correlation between the usDOI and pDOI, we can indirectly assume that the usDOI may be a preoperative prognostic factor for nodal disease: future studies must be conducted to support this hypothesis.

## 5. Conclusions

In conclusion, our study showed a good agreement between the usDOI and pDOI. We believe that an IOUS might be indicated for the local staging of early clinical N0 oral lesions: this is the scenario in which the precision in estimating the DOI would have a greater clinical impact, at least to estimate the risk of occult lymph node metastases. In contrast, MRI might be proposed for more advanced lesions or if suspicious or enlarged lymph nodes are present, given its panoramic view and better sensitivity in detecting bone invasions and floor-of-the-mouth infiltration. However, larger and prospective studies must be conducted to confirm this hypothesis.

## Figures and Tables

**Table 1:** Advantages and disadvantages of computed tomography (CT), magnetic resonance imaging (MRI), and intraoral ultrasound (IOUS) in oral cancer local and regional staging.

	Advantages	Disadvantages
*CT*	Spatial resolution Visualization of bone erosion Panoramic	Low contrast resolution Influenced by metal artifacts Superficial lesions not always visible
*MRI*	Contrast resolution Multiparametric Panoramic	Influenced by metal artifacts Superficial lesions not always visible
*IOUS*	Very high spatial resolution Direct visualization of lesions	Operator dependent: rotational overestimation, compression distortion, need for expert hands Not possible if trismus is present Expensive and not diffuse technology

**Table 2:** Clinicodemographic data of patients included in the study.

	*N* (%)
*Patients*	**41 (100)**
*Females*	**16 (39)**
*Males*	**25 (61)**
*Mean age (st. deviation)*	**64.07 (17.67)**
*pTis*	**5 (12.20)**
*pT1*	**21 (51.22)**
*pT2*	**15 (36.58)**
*Mean pDOI (st. deviation)*	**3.07 (2.65)**
*Mean usDOI (st. deviation)*	**3.79 (2.74)**
*Mean mriDOI (st. deviation)*	**5.39 (2.81)**

## Data Availability

Not applicable.
